# Comparative Study on Postoperative Immobilization in Reverse Total Shoulder Arthroplasty: 4 Weeks vs. 6 Weeks of Immobilization Yields Similar Clinical and Functional Outcomes

**DOI:** 10.3390/jcm13216363

**Published:** 2024-10-24

**Authors:** Felix Hochberger, Moritz Friedrich Wild, Tizian Heinz, Maximilian Rudert, Kilian List

**Affiliations:** Department of Orthopaedic Surgery, Julius-Maximilians University Wuerzburg, Koenig-Ludwig-Haus, Brettreichstrasse 11, 97074 Wuerzburg, Germany; felix.hochberger@klh.de (F.H.); moritz.wild@klh.de (M.F.W.); tizian.heinz@klh.de (T.H.); maximilian.rudert@klh.de (M.R.)

**Keywords:** osteoarthritis of the shoulder, rehabilitation, cuff tear arthropathy, postoperative treatment, functional outcomes, immobilization, complication rate

## Abstract

**Background/Objectives**: To investigate clinical and functional outcomes of patients undergoing reverse total shoulder arthroplasty (RTSA) using a rehabilitation protocol of either 4 or 6 weeks of immobilization. **Methods**: This comparative study analyzed a consecutive patient cohort that underwent RTSA in a single institute from January 2021–February 2023. Patients were assigned to groups according to the duration of postoperative immobilization and were followed up for a minimum of one year. Patient demographics, range of motion (ROM), functional outcomes using the Simple Shoulder Test (SST) and the American Shoulder and Elbow Score (ASES) as well as postoperative complications were recorded. The minimum clinically important difference (MCID) was used to assess whether the differences between the groups in SST, ASES, SWB, and VAS were clinically relevant. **Results**: Sixty patients met the inclusion criteria (35 patients in the 6-week immobilization group (6 WG) and 25 patients in the 4-week immobilization group (4 WG)) and were available for the total follow-up period. Similar baseline demographics were observed between the groups. Active ROM significantly improved for both groups, with abduction and forward flexion improving the most. In terms of functional outcomes, significant improvement (*p* < 0.001) was demonstrated for both groups (ASES, SST, VAS, and SWB). However, patients in the 4 WG reached significantly better results in VAS and SWB at 6 weeks and 3 months, as well as in ASES 3 months postoperatively, whereas both groups leveled off at the end of the follow-up period after 1 year. Taking into account the MCID, these differences for ASES at 3 months, as well as VAS and SWB at 6 weeks and 3 months postoperatively, were significant. Surprisingly, there were no differences between the groups over the entire follow-up period in terms of the SST. **Conclusions**: The author’s findings suggest faster clinical recovery at 6 weeks and 3 months in patients undergoing postoperative immobilization of 4 weeks compared to 6 weeks following RTSA. However, clinical and functional outcomes were equal for 4 WG and 6 WG at one year follow up.

## 1. Introduction

Reverse shoulder arthroplasty (RTSA), introduced by Paul Grammont in 1985, has progressively gained popularity as a treatment option for a wide range of shoulder pathologies [1]. Initially developed for managing rotator cuff arthropathy, its indications have expanded over time due to consistently positive clinical outcomes and high patient satisfaction. Today, RTSA is also employed to treat conditions such as shoulder osteoarthritis, proximal humeral fractures in elderly patients, neoplasia, refractory glenohumeral instability, irreparable rotator cuff tears, and pseudoparalysis [2,3]. The procedure has been shown to effectively alleviate pain and improve shoulder function, making it a reliable option for restoring activities of daily living [1]. Over the years, advancements in implant design, surgical techniques, and rehabilitation protocols have aimed to further minimize complications and enhance clinical outcomes. As a result, RTSA has remained a focal point of orthopedic research in recent decades. However, data on standardized postoperative rehabilitation protocols are still limited due to a substantial divergence of available studies in terms of the duration of postoperative immobilization, initiation of active range of motion, and muscle activation exercises [4,5,6,7]. Most of these studies are based on anatomical and biomechanical knowledge [4,6,7], without considering clinical and functional results on complication rates. Postoperative immobilization was implemented to ensure implant integration and promote soft tissue healing. The main concern with early active rehabilitation and a shorter postoperative immobilization period is the potential risk of postoperative instability of the prosthesis and acromial overload due to deltoid tension, which may theoretically increase the likelihood of a stress reaction or stress fracture [8,9,10]. However, prolonged immobilization appears to negatively impact postoperative range of motion and muscle strength, particularly in elderly patients, potentially resulting in poor functional outcomes [11]. A comprehensive systematic review carried out by Bullock et al. in 2019 examined the literature for suitable studies and identified substantial discrepancies between authors on the optimal duration of protection and progression of rehabilitation and activity [12]. To address this gap in literature, this study was designed to assess functional outcomes, range of motion, as well as complication rates following RTSA, contingent upon the duration of postoperative immobilization (4 vs. 6 weeks). The authors hypothesized that, compared to a postoperative immobilization period of 6 weeks, a 4-week immobilization period will lead to comparable functional outcomes and range of motion, high patient satisfaction, and no increase in complications.

## 2. Materials and Methods

### 2.1. Patient Selection

A retrospective comparative study was conducted to evaluate patients who underwent RTSA for postoperative rehabilitation protocols between April 2017 and February 2023 at the authors institution. Patients were included for any indication leading to implantation of an RTSA except for revision cases, aged over 18 years of age and available for a postoperative follow-up of 6 weeks and 3 and 12 months. We excluded patients with prior arthroplasty procedure to the affected shoulder as well as patients with neuromuscular diseases. The in-house rehabilitation procedure for the duration of postoperative immobilization following RTSA was changed from 6 to 4 weeks by January 2021. The clinic’s own database was analyzed to identify eligible patients. Group allocation (4 WG and 6 WG) was determined by the duration of postoperative immobilization, as recorded in the surgical reports. An a priori power analysis was conducted using G*Power version 3.1 (Düsseldorf, Germany) to determine the necessary sample size for the primary outcome variable, the American Shoulder and Elbow Surgeons (ASES) score. The analysis indicated that a total of 62 shoulders (31 per group) were required to detect a minimum clinically important difference (MCID) of 13.6 points on the ASES score, with a significance level of *p* < 0.05 and 80% statistical power. Ethics approval at the author’s institution was obtained (Ref. 2024012901).

### 2.2. Surgical Management

**Figure 1:**
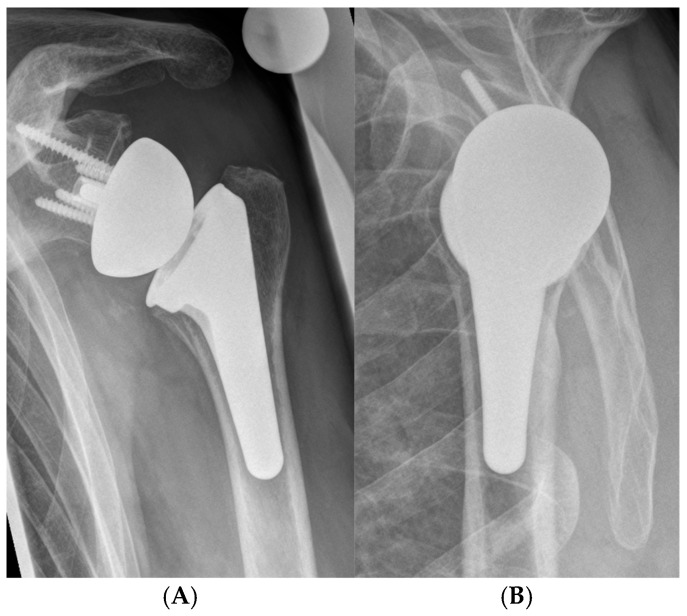
Postoperative radiograph showing a cementless short stem reverse shoulder arthroplasty in a.p. (**A**) and y-view (**B**).

All operations were performed by one fellowship-trained senior consultant surgeon (K.L.) using the department’s standard implants (Tornier Humeral Perform or Ascend Flex (humeral side) and Aequalis Revers 2 or Perform Revers (glenoid side), Fa. Stryker, Bloomington, MN, USA) (Figure 1) with the patient placed in beach-chair position. A standard deltopectoral approach was used in all cases. Tenotomy and repair of the subscapularis tendon was performed in all of the cases, if tendon quality was sufficient. Additional bony increased offset (BIO)-RTSA was performed (with a Aequalis revers 2, Fa. Stryker, Bloomington, MN, USA) in case of sufficient humeral bone graft. Metal augments were used if no graft was available (Fa. Stryker, Bloomington, MN, USA). In rare cases of severe osteopenia or substantial metaphyseal bone loss the humeral component was cemented. All patients had preoperative images in a.p., axial and outlet view and postoperative images in a.p. and axial view at 4 weeks and 1 year after surgery. The vast majority of procedures were planned with a 3D planning software (Blueprint, Fa. Stryker, Bloomington, MN, USA).

### 2.3. Postoperative Rehabilitation

**Figure 2:**
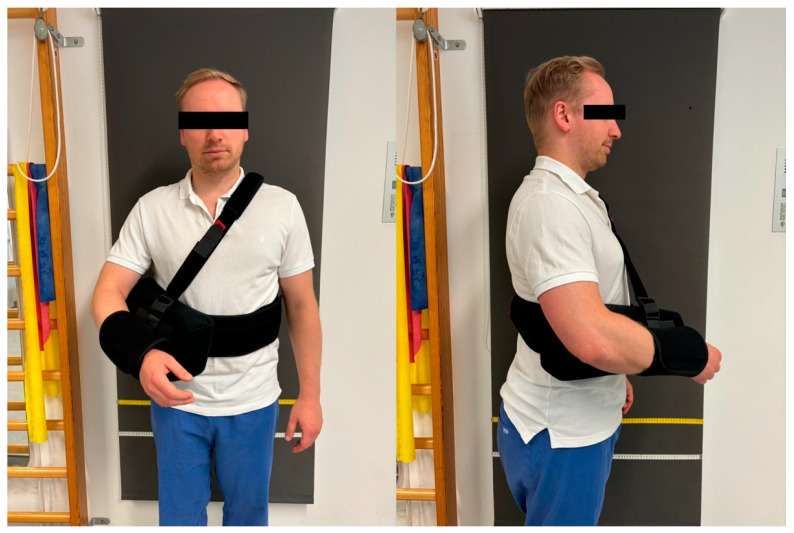
Figure demonstrating postoperative immobilization in an abduction sling after RTSA.

**Figure 3:**
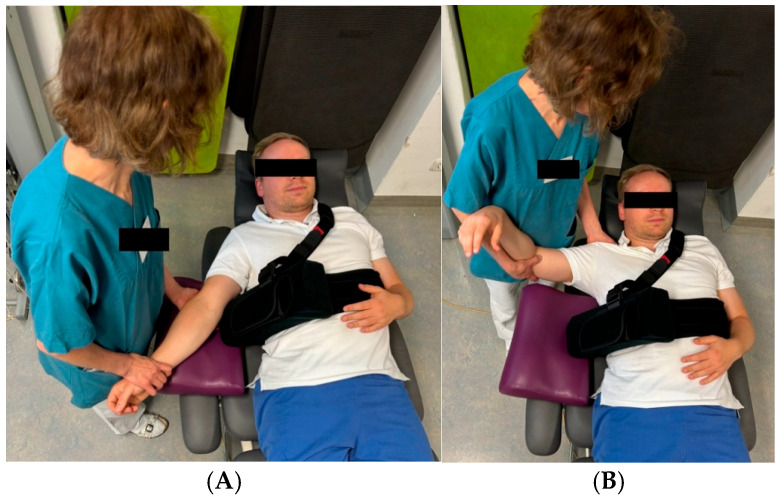
Figure demonstrating immediate postoperative mobilization of a patient who has undergone RTSA by a physiotherapist. (**A**) Passive motion of the adjacent joint. (**B**) Passive abduction up to 90°.

The postoperative rehabilitation protocol for the 6-week group (6 WG) was carefully structured to promote optimal recovery while minimizing complications. During the first two weeks postoperatively, patients were immobilized in a shoulder brace with a pillow (Figure 2), to be worn continuously both day and night. Passive range of motion exercises were restricted to forward flexion up to 90°, external rotation up to 10°, and abduction up to 60°. Close attention was given to maintaining proper scapular movement, and isometric exercises for the deltoid muscle were introduced early in the process. Figure 3 illustrates the immediate postoperative mobilization of a patient following RTSA. Gradual removal of the orthosis commenced after two weeks, with complete discontinuation by the end of the sixth week. Beginning in week 7, patients progressed to unrestricted passive range of motion and gradually initiated active exercises, including gentle stretching of the deltoid and subscapularis muscles. By week 9, resistance exercises with light weights were introduced, particularly targeting the deltoid muscle for strengthening. The 4-week group (4 WG) followed an accelerated rehabilitation timeline. The stepwise removal of the orthosis began two weeks earlier than in the 6 WG, with full discontinuation by the end of the fourth week. The transition from passive to active range of motion exercises was initiated at week 5, allowing for earlier functional recovery. Additionally, gentle strengthening exercises for the deltoid muscle were incorporated from week 7, facilitating earlier muscle conditioning. Both postoperative rehabilitation protocols were meticulously developed in collaboration with senior chief surgeons of the shoulder and elbow department and the clinic’s lead physiotherapists. This collaborative approach ensured that each protocol balanced the need for controlled healing with the promotion of functional recovery, providing a comprehensive framework to minimize complications and optimizing clinical outcomes.

### 2.4. Outcome Evaluation

Data were obtained 4 or respectively 6 and 12 weeks postoperatively and at the end of the follow-up period (after 12 months). Clinical and functional status across the follow-up check points were reproduced from outpatient clinic reports and the rehabilitation report. Surgery-specific details such as fixation technique (cemented vs. non-cemented), implant type and additive BIO-RTSA were extracted from the corresponding surgery report. Functional outcome was assessed using the American Shoulder and Elbow Score (ASES) and the Simple Shoulder Test (SST). Pain status was evaluated using the visual analog scale (VAS) with a rating range of 0–10. 0 was defined as pain-free and 10 as the most severe pain imaginable. The degree of patient satisfaction was evaluated on a scale of 4 levels: 0: highly satisfied, 1: largely satisfied, 2: rather unsatisfied, and 3: unsatisfied. Clinical assessment included measurement of active range of motion of the affected shoulder using a goniometer. The minimum clinically important difference (MCID) was used to assess whether the differences between the groups in SST, ASES, SWB, and VAS were clinically relevant. Outcome evaluations and physical examinations were performed by two examiners who were not involved in the surgical treatment (M.W. and F.H.). Demographic data such as age, gender, occupation, and sports activities were additionally obtained.

### 2.5. Statistical Analysis

All analyses were performed using SPSS Statistics (version 29, owned by IBM Corp., Armonk, NY, USA). Descriptive statistics were used for continuous variables. Normal distribution was analyzed using the Shapiro–Wilk test and normally distributed values were described by mean and standard deviation. In cases of non-parametric distributions, the median and interquartile range (Q0.25–Q0.75) was used to describe the data. If a normal distribution was present, a student *t*-test was applied. For non-parametric variables, a Mann–Whitney U-test was used to compare outcomes between the two groups. For the intragroup analysis, a repeated measures ANOVA or Friedman-test was employed where applicable. Statistical significance was set at *p* < 0.05.

## 3. Results

### 3.1. Patient Demographics

**Figure 4:**
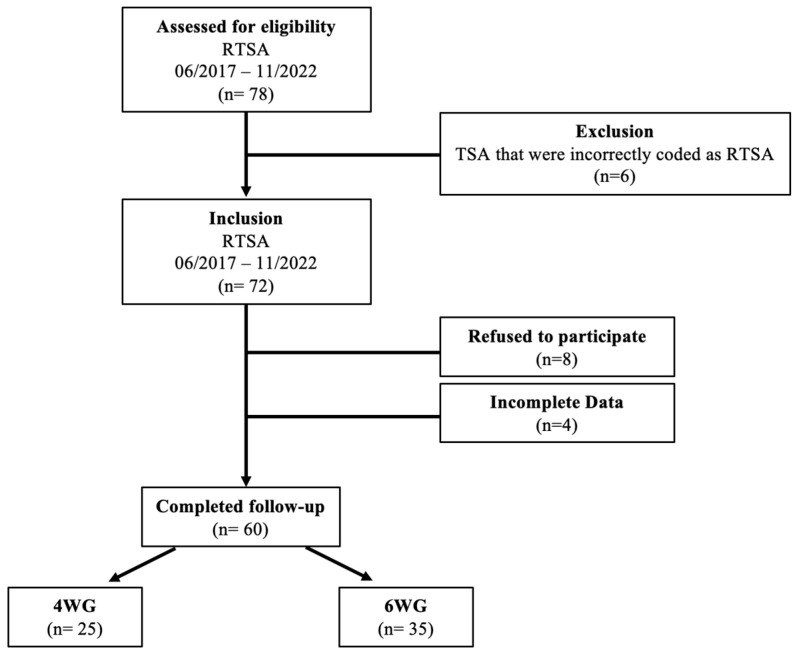
Flowchart displaying patients meeting study criteria.

In the author’s department, postoperative immobilization following RTSA was changed from 6–4 weeks in January 2021. Postoperative clinical-radiological follow-up examinations were regularly carried out 4 or, respectively, 6 weeks and 1 year after surgery. Out of these, a smaller cohort was scheduled for a 3 months follow-up appointment after completing inpatient rehabilitation. To make the most accurate statements about changes in clinical and functional status in the postoperative process, this study only included patients who appeared at all three follow-up time points (4 or, respectively, 6 weeks, 3 months, and 1 year postoperatively). Based on this approach, analysis of the internal database revealed a total of 78 patients who underwent RTSA between June 2017 and November 2022 and met the inclusion criteria as mentioned above. A review of surgical reports revealed that six patients had to be subsequently excluded because they had been treated with TSA and were incorrectly coded as RTSA. Moreover, eight patients refused to participate in the study and four patients showed incomplete data, leaving a total of 60 patients who completed the whole follow-up. Of these, 35 patients were allocated to the 6 WG and 25 patients to the 4 WG. The patients who met the inclusion criteria a demonstrated in Figure 4. Patient demographics are presented in Table 1.

### 3.2. Clinical Results and Functional Outcomes

In terms of range of motion, both groups showed comparable improvements in flexion, abduction, external rotation, and internal rotation over time, as demonstrated in Table 2.

**Figure 5:**
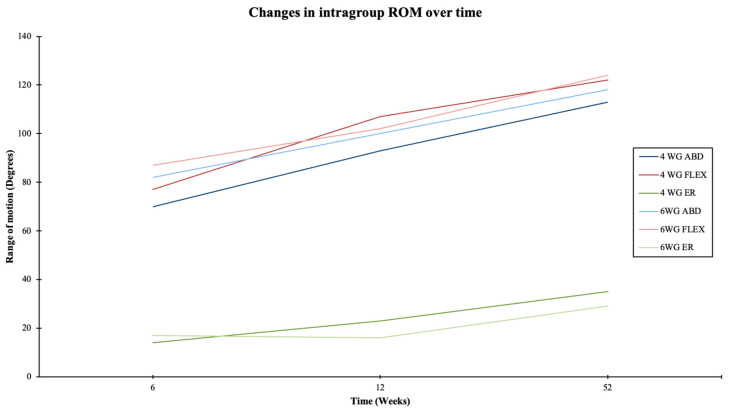
Graphic demonstrating changes in range of motion over the follow-up period within the groups.

Significant changes in intragroup ROM were noted for all directions over the follow-up period in both groups, as demonstrated in Table 3 and Table 4 as well as in Figure 5.

Active abduction in the 4 WG was initially slightly lower at 70 ± 23° compared to 81 ± 20° in the 6 WG at the 6-week follow-up, although this difference was not statistically significant (*p* = 0.061). By 3 months, abduction in the 4 WG improved to 93 ± 27° (*p* < 0.001) and reached 113 ± 37° at 1 year postoperatively, while the 6 WG showed similar improvements to 100 ± 26° and 118 ± 30° over the same periods (*p* < 0.001). A similar pattern was observed in flexion, with the 6 WG initially demonstrating a slight advantage (87 ± 20° vs. 77 ± 23°, *p* = 0.229), but both groups showed significant improvements by 1 year (4 WG: 122 ± 31°, 6 WG: 124 ± 29°, *p* < 0.001). In terms of external rotation, the 4 WG exhibited greater improvement (from 14 ± 11° to 35 ± 9°) compared to the 6 WG (from 17 ± 12° to 29 ± 14°, *p* = 0.295). Internal rotation improved similarly in both groups, with the 6 WG initially showing a slight advantage (*p* = 0.858). Regarding functional status, all assessed scores (ASES, SST, VAS, and SWB) in the 6 WG showed significant improvements across all three follow-up points (*p* < 0.001). The 4 WG also showed significant improvements, except for VAS and SWB, where no significant differences were observed between the follow-up periods (VAS: *p* = 0.291, SWB: *p* = 0.107). In the 4 WG, 84% of patients achieved a VAS of 0 at 1 year, while 2 patients experienced postoperative dislocations that did not require revision surgery. Intergroup comparison showed that the 4 WG had better VAS scores at 6 weeks (*p* = 0.001) and 3 months (*p* = 0.012) postoperatively, as well as better ASES scores at 3 months (*p* = 0.023). Subjective well-being (SWB) was significantly better in the 4 WG at 6 weeks (*p* = 0.007), with differences persisting until 3 months, but leveling out by 1 year (*p* = 0.831). Please see Table 5 and Table 6.

Complications were rare and occurred at a comparable rate between the groups, including nerve irritations and a required revision surgery in the 4 WG, as well as early aseptic loosening in the 6 WG.

## 4. Discussion

The current study revealed that a shortened postoperative immobilization of 4 weeks does not increase the complication rate and offers faster recovery in the short term, with comparable clinical results after 1-year follow-up. Patients of the 4 WG achieved significantly better results in terms of VAS and SWB 6 weeks postoperatively as well as in ASES 3 months postoperatively, which were considered to be clinically significantly according to the MCID [13,14,15,16]. One patient of the 4 WG reported subluxation events and loss of active external rotation leading to revision surgery with liner exchange and L’Episcopo tendon transfer. Another patient of the 4 WG reported prolonged postoperative disorder due to irritation of the suprascapular nerve caused by a glenoid screw with full recovery at the 1-year follow up. However, this complication was not linked to the rehabilitation protocols investigated in this study and therefore was excluded from the analysis. One patient in the 6 WG showed early aseptic loosening of the stem at the 1-year follow-up, which also led to surgical revision with replacement of the glenoid component. Both groups showed very low complication rates with no differences in intergroup comparison.

To a large extent, strategies for postoperative rehabilitation following RTSA still vary widely, and standardized guidelines have not yet been established. A systematic review from 2019 analyzed the available literature for published studies on postoperative treatment strategies after shoulder arthroplasty. The authors could only identify six studies investigating post-RTSA procedures [12]. The authors reported that the results of various shoulder surgeons with their publications on RTSA postoperative management included significant variability in the duration of sling treatment and the initiation of active range of motion. However, all protocols concurred on the importance of initiating deltoid isometric exercises soon after surgery [5,6,7,17,18,19]. All posttreatment protocols more or less aim to avoid combined extension, adduction and internal rotation in order to prevent instability and create scar formation around the center of rotation around the glenohumeral joint [5,6,7,17,18,19]. These programs mainly differ in the timepoint as to when these movements are allowed after surgery. A notable level of heterogeneity exists in the rehabilitation guidelines and associated precautions for both TSA and RTSA. As most of these protocols are based on biomechanical findings, the authors concluded that there is a need to determine optimal rehabilitation approaches after TSA and RTSA based on clinical outcomes [12]. To our knowledge, this is one of only a few studies investigating clinical efficacy of two different posttreatment protocols of patients undergoing RTSA and the first to assess those results, taking into account the MCID.

In 2021, the research group led by Edwards et al. conducted a prospective randomized study to investigate if an early, active mobilization regimen focusing on the deltoid and external rotator muscles might result in superior postoperative outcomes compared to a delayed mobilization program primarily targeting the deltoid muscle following RTSA [20]. Like Hagen et al. [11], the authors could demonstrate that early- and delayed-rehabilitation programs may result in significant improvements in ROM and PROMs. However, in contrast to Hagen et al., both of the two rehabilitation protocols applied were initiated faster. The delayed group in Hagen’s study was prohibited from any exercise until the sixth postoperative week, whereas in Edward’s delayed group was prescribed PROM exercises from the second postoperative week. Additionally, submaximal isometric exercises were started in the early mobilization group from the second postoperative week and external rotation against resistance from the sixth postoperative week, whereas Hagen et al. only started these exercises after 6 and 12 weeks, respectively. These findings may be explained by the results of Denard and Laderman et al. in 2016 [21]. They explored the effects of “immediate” versus “delayed” passive and active-assisted range of motion (ROM) exercises in a cohort undergoing anatomic total shoulder arthroplasty. They found no discernible differences in ROM and functional outcomes at 3, 6, and 12 months post-surgery [21]. In our study, there was a continuous improvement in the different directions of active range of motion in both groups with subjectively satisfactory results after 1 year. These findings differ from these of Hagen and Edwards et al., who both demonstrated either improvements in external rotation and a delay in internal rotation from three months post-surgery [20] or no improvement in external rotation in either the immediate or delayed rehabilitation group [11]. This improvement in ROM is particularly crucial for patients in performing daily activities, such as toileting, washing the back, and, notably for women, manipulating and adjusting a bra strap. Considering the literature, the results for postoperative internal rotation after 1 year in both groups of our study (4 WG IR 1 year: 85 ± 16; 6 WG IR 1 year: 91 ± 14) seem to be favorable and comparable to those obtained in previous studies [22]. Following RTSA, a major goal is to gradually regain overhead motion, with previous studies demonstrating a correlation between deltoid strength and active arm flexion [23,24]. It is widely recognized that targeting the deltoid, particularly its anterior head, is crucial in rehabilitation post-RTSA, given its primary role in arm flexion [25]. Although many rehabilitation protocols acknowledge the importance of deltoid strengthening post-RTSA, several adopt a conservative approach in their recommendations due to concerns regarding scapular notching, acromial stress fractures, and implant loosening [12]. In a systematic review of rehabilitation protocols, Bullock et al. reported that only three [6,18,19] out of six protocols advised early deltoid isometrics within the initial six weeks following RTSA, with the recommendation for achieving active motion by 12 weeks post-surgery.

It is known that postoperative instability is one of the major complications following RTSA, which occurs in approximately 2, 4–31% [26] of cases and predominantly appear within the first 3 months postoperatively [26,27,28]. To avoid instability, a more restrained rehabilitation protocol with prolonged immobilization and delayed active range of motion appears to be beneficial. However, this in turn may negatively affect postoperative clinical and functional outcomes and therefore promotes an early pro-active approach. Studies on this topic refer to the complexity of finding the balance between early active mobilization, delta activation, and immobilization in the sling [6,7,12]. In their retrospective comparative study from 2017, Romano et al. investigated various personalized posttreatment protocols for patients undergoing RTSA regarding their effectiveness and complication rate [6]. Based on clinical, radiological and intraoperative criteria, three groups (“low-care”, “medium-care”, and “high-care”) were established, each with a specific level of postoperative care. They found statistically significant improvements in all parameters analyzed compared to the preoperative assessment, with remarkable improvements reported especially in the “high-care” group. There was only one shoulder dislocation (0.8%) in the “high-care” group, and they concluded that it would be difficult to establish universal parameters for a rehabilitation protocol and that the implementation of personalized protocols as well as early discharge from the immobilization sling can significantly improve clinical outcomes at one year follow-up and may reduce complication rates [6]. Moreover, the authors emphasized the importance of physiotherapeutic management of the scapulothoracic setting in order to avoid possible impingement by transferring part of the glenohumeral range of motion of the humerus to the scapulothoracic joint [29]. Similar findings were made by Hagen et al. in 2019 [11]. In the study presented here, not a single shoulder dislocation occurred in the 6 WG during the postoperative follow-up, whereas one patient from the 4 WG developed early instability, which resulted in surgical revision after failed conservative treatment. Although only 1-year results are presented, this postoperative complication rate is in line with the current literature [9,30]. Since rehabilitation-related complications are to be expected within this first year after surgery and the 1-year outcome is regarded merely as an endpoint for the final postoperative success, we consider 1-year outcomes to be sufficient.

In our study, adherence to rehabilitation protocols by patients and physiotherapists was not systematically recorded, which is a limitation when fully assessing its impact on postoperative outcomes. Compliance with rehabilitation is widely recognized as a crucial factor influencing recovery following RTSA [31]. Variability in adherence could potentially affect functional outcomes, complication rates, and overall patient satisfaction. Although we could not analyze compliance in detail due to the retrospective nature of our study, we do not believe that the duration of sling therapy (4 or 6 weeks) significantly influenced compliance.

As part of the postoperative stability of an RTSA, the role of the subscapularis tendon is still controversially discussed. Romano et al. performed a refixation of the tendon whenever tissue conditions were favorable, which is consistent with the approach in the study presented here based on the assumption that refixation of the subscapularis may increase anterior stability of the implant in deltopectoral approach surgery by increasing the thickness of the soft tissue between the skin and prosthesis [32,33,34]. Vourazeris et al. [33] and Clark et al. [9] reported no significant disparity in dislocation rates and complication incidence between patients undergoing subscapularis repair and those without. Conversely, Edwards et al. [20] concluded that none of their patients, who experienced implant dislocation, had undergone subscapularis tendon repair. In our study, patients of both groups underwent refixation of the subscapularis tendon.

In the context of our study, the transition from the Aequalis Ascend Flex to the Tornier Humeral Perform implant did not result in significant differences in clinical or functional outcomes. Both implants were used following consistent surgical principles, particularly regarding inclination (within 2.5 degrees) and metaphyseal fixation, minimizing the likelihood of any notable impact on the parameters investigated. However, we recognize that a more detailed comparison between these two implants could provide further insights, especially in terms of complication rates, surgical duration, and long-term functional outcomes. Although our data show no significant variations between implant types, the lack of notable differences may be attributed to the relatively small sample size and the retrospective nature of our analysis. Future studies with larger cohorts and a prospective design should further explore the potential influence of different humeral implants on postoperative outcomes. Specifically, a targeted analysis focusing on complication rates, functional recovery, and long-term implant durability may provide more definitive conclusions regarding the comparative performance of these devices. This is particularly relevant as implant designs continue to evolve, potentially introducing subtle variations in biomechanical properties and clinical performance that were not captured in our study. Therefore, future research comparing these implant types could contribute to improved surgical decision making and better patient outcomes.

In our study, we found no significant differences in sex distribution between the two groups (*p* = 0.777). To investigate whether sex could impact complication rates, we conducted further analysis. Our findings indicated that gender did not significantly influence the occurrence of complications. These results partially contradict existing literature. A recently published systematic review by Bindi et al. [35] identified male sex as a significant risk factor for postoperative instability following reverse total shoulder arthroplasty (RTSA). Additionally, a large multi-center study reported that male patients had a 2.2-fold increased risk of postoperative instability after RTSA [36], with other studies suggesting an even higher risk in men, ranging from 3–8 times greater [37,38,39]. The likely explanation for this increased risk in male patients is their tendency to engage in higher levels of physical activity, placing greater and more consistent stress on the implant. The discrepancy in our study’s findings may be attributed to the smaller sample size.

Between the two groups, a similar rate of stem cementation could be observed with no significant difference (*p* = 0.454). Due to the fact that the RTSA procedures with a 6-week postoperative immobilization was performed at an earlier stage than those with a 4-week postoperative immobilization, it could be assumed that knowledge about the benefits of stem cementation would have changed over time. However, a review of the current literature shows the opposite [40,41,42,43]. Modern implants with improved cementless metaphyseal fixation options as well as challenging intraoperative revision conditions associated with stem replacement have led to the recommendation that cementing of the stem should only be performed in revision cases, in cases of severe osteoporosis or in highly aged patients [42,43]. Moreover, there is evidence that modern metaphyseal short-stem prostheses provide good to excellent long-term results in terms of durability and revision rates without increased stem-related osteolysis compared to cemented stems [44]. Furthermore, the type of stem fixation is known to have no influence on postoperative instability [37,45,46]. With a slight superiority of an 88% press-fit fixation rate in the 4 WG (80% in the 6 WG), the results of the present study are consistent with the trends in the current literature.

Patients of both groups in this study were able to waive a considerable reduction in their pain perception based on the VAS over the entire follow-up period; however, it should be noted that no preoperative score was recorded. Interestingly, the 4 WG demonstrated practically complete pain relief after only 6 weeks. This difference between the groups was significant and considered to be clinically significant according to the MCID (change of 1.3–3/10) [13,15]. VAS values levelled off at the 1-year follow-up examination. Previous studies indicate that patients undergoing RTSA typically experience substantial pain reduction and can achieve most of their functional improvements within six months postsurgery, with maximum improvement typically reached by 12 months [47,48]. This also explains the steady improvement in functional status of patients in both groups according to the ASES and SST. In contrast to the findings of Simovitch et al. [49], we were further able to report improvements in ASES and SST in both groups between 6 months and 1 year postoperatively. Interestingly, patients from the 4 WG showed significantly better ASES scores at 3 months compared to the 6 WG. Considering the baseline ASES scores of both groups we observed a notable increase of approximately 30 points in the ASES score at the one-year mark postsurgery (ASES 4 WG 6 weeks: 54, 1 year: 90; ASES 6 WG 6 weeks: 57, 1 year: 83). These findings align closely with results reported in several studies [50,51,52] and is slightly lower than the improvements noted in others, which ranged from 40–45 points [53,54]. The observed approximate 30-point improvement surpasses the MCID range of 6–15 points [14,15,16] and is, therefore, considered to be clinically significant. Patients from the 4 WG achieved significantly better outcomes 6 weeks and 3 months postoperatively than those from the 6 WG based on the defined SWB criteria. This is likely due to the significant better outcomes in terms of VAS at 6 weeks as well as VAS and ASES at 3 months postoperatively. Despite the fact that no standardized SWB criteria for patients undergoing RTSA has been described so far, SWB in patients with rotator cuff (RC) tears has been linked to pain, dysfunction, functional challenges, and postoperative outcomes following RC surgery [55,56].

The current study contributes valuable data to understanding the necessity of postoperative immobilization. It partially contradicts claims that early rehabilitation may promote faster functional recovery, while conservative protocols could minimize complications [31]. We have included our institutional rehabilitation protocol to encourage other research groups to contribute to this important area of study. We emphasize the importance of comprehensive informed consent, where physicians should discuss the risks, benefits, and alternatives of each rehabilitation strategy, ensuring patients are fully informed before making treatment decisions [57].

This study is not without limitations. The retrospective nature of this study should be acknowledged. In our study, the one-year follow-up was sufficient to capture early postoperative complications and improvements in functional outcomes. Although it seems unlikely that complications occurring more than a year after surgery are related to the rehabilitation protocol, the current data cannot conclusively prove this. Another limitation is the absence of preoperative functional and pain data, which restricts our ability to make precise comparisons between preoperative and postoperative states. Including assessments such as the VAS for pain, SST, and ASES scores for function would have allowed a more comprehensive evaluation of postoperative improvements. As a result, this limitation may affect the interpretation of the degree of functional recovery and pain relief observed in our study. Nonetheless, the focus of this study was on complication rates and the pace of clinical improvement. The homogeneity of the groups and strict inclusion and exclusion criteria have produced a highly valuable dataset for evaluating the postoperative healing process. During the period under review the predominately used humeral implant changed (until November 2022 Aequalis Ascend Flex followed by Tornier Humeral Perform, Fa. Stryker, Bloomington, MN, USA). The glenoid side was not changed. Both implants are short stem implants, in the majority non-cemented metaphyseal fixated with an inclination of 142.5 and 145°, respectively. Within the accuracy of implantation, we assume the inclination and therefore no effect on the investigated parameters. Furthermore, the compliance of the patients and the treating physiotherapists regarding the prescribed rehabilitation protocol may have varied.

## 5. Conclusions

The results of this study suggest that a postoperative rehabilitation strategy including immobilization of 4 weeks following RTSA leads to comparably favorable clinical and functional results as of 6 weeks while offering faster clinical recovery at 6 weeks and 3 months. Further prospective randomized studies investigating the necessity of postoperative immobilization in its entirety would be advisable.

## Figures and Tables

**Table 1:** Patient demographics between the 4- and 6-week groups. Data are shown as numbers (percent) or median ± interquartile range, with Mann–Whitney U test, except when denoting a normal distribution, as was applicable for age in years. Here, the variable was written in cursive and the mean, with standard deviation, and *t*-test was employed.

Parameter		4 WG			6 WG			*p*-Value
		n (%)	Mean/ Median	±SD/ IQR	n (%)	Mean/ Median	±SD/ IQR	
Age (years)			59.7	7.0		75.5	5.7	0.198
Sex								0.777
	Male	7 (28.0)			11 (31.4)			
	Female	18 (72.0)			24 (68.6)			
BMI (kg/m^2^)			28.5	4.4		27.4	7.8	0.828
Diagnosis								0.535
	CTA	10 (40.0)			19 (54.3)			
	OA	13 (52.0)			11 (31.4)			
	Pathological fracture	1 (4.0)			1 (2.9)			
	Chronic instability	0 (0.0)			2 (5.7)			
	Humeral head necrosis	0 (0.0)			2 (5.7)			
	Infection	1 (4.0)			0 (0.0)			
Implant type								0.103
	RTSA	5 (20.0)			14 (40.0)			
	BioRTSA	20 (80.0)			21 (60.0)			
Side								0.862
	Left	12 (48.0)			16 (45.7)			
	Right	13 (52.0)			19 (54.3)			
Fixation								0.454
	Cemented	3 (12.0)			7 (20.0)			
	Press-fit	22 (88.0)			28 (80.0)			

Abbreviations: 4 WG, 4-week-group; 6 WG, 6-week-group; n, number, SD, standard deviation, IQR, interquartile range; RTSA, reverse total shoulder arthroplasty; BioRTSA, bony increased offset reverse total shoulder arthroplasty; OA, primary osteoarthritis of the shoulder; CTA, cuff tear arthropathy.

**Table 2:** Active range of motion in degree at follow-up. Data are shown as mean ± standard deviation/interquartile range, with Mann–Whitney U test, except when denoting a normal distribution. In these cases, the variable was written in cursive and the mean, with standard deviation, and *t*-test was employed. Significance denoted by *. Abbreviations: ABD, abduction; FLEX, forward flexion; ER, external rotation; IR, internal rotation; ASES, American Shoulder and Elbow Surgeons Shoulder score; SST, Simple Shoulder Test; VAS, Visual Analog Scale; SWB, subjective well-being.

	4 WG		6 WG		*p*-Value
6 Weeks Postop	Mean/Median	±SD/IQR	Mean/Median	±SD/IQR	
ABD	80	30	90	20	0.061
FLEX	80	30	90	20	0.229
ER	10	15	20	20	0.295
IR	80	43	80	0	0.858
ASES	54	10	57	11	0.295
SST	2	1	2	2	0.067
VAS	0	2	2	2	0.001 *
SWB	0	1	1	1	0.007 *
3 months postop					
ABD	90	20	90	20	0.301
FLEX	110	35	95	20	0.311
ER	20	28	20	10	0.157
IR	80	30	85	10	0.264
ASES	65	13	57	12	0.023 *
SST	4	5	3	2	0.698
VAS	0	2	2	1	0.012 *
SWB	0	1	1	0	0.007 *
1 year postop					
ABD	113	37	118	30	0.327
FLEX	122	31	124	29	0.390
ER	30	13	30	20	0.054
IR	90	0	90	20	0.337
ASES	90	19	83	12	0.071
SST	9	6	7	2	0.093
VAS	0	0	0	1	0.335
SWB	0	1	0	1	0.831

**Table 3:** Intragroup Changes (6 WG) in active range of motion over the follow-up period. Data are shown as mean ± standard deviation. ANOVA test for repeated measures was performed. If the Mauchly test was valid (*p* > 0.05), sphericity was assumed and the Mauchly *p*-value was used. In 4 WG, Mauchly test was valid for all directions. If the Mauchly test was violated (*p* < 0.05), an additional Epsilon test was assessed. If the Epsilon was greater than 0.75, the Greenhouse–Geisser correction was used to assess for significance (*p* < 0.05). Abbreviations: SD, standard deviation; FU, Follow-Up; ABD, abduction; FLEX, forward flexion; ER, external rotation; IR, internal rotation.

6 WG		Mean	SD	Mauchly	*p*-Value	Epsilon	Greenhouse–Geisser *p*-Value
Direction	FU						
ABD				0.034		0.883	<0.001
	6 weeks	82	20				
	3 months	100	26				
	1 year	118	30				
FLEX				0.011		0.843	<0.001
	6 weeks	87	18				
	3 months	102	19				
	1 year	124	29				
ER							
	6 weeks	17	12	0.248	<0.001		
	3 months	16	9				
	1 year	29	14				
IR							
	6 weeks	78	13	0.154	<0.001		
	3 months	80	15				
	1 year	91	14				

**Table 4:** Intragroup Changes (4 WG) in active range of motion over the follow-up period. Data are shown as mean ± standard deviation. ANOVA test for repeated measures was performed. If the Mauchly test was valid (*p* > 0.05), sphericity was assumed and the Mauchly *p*-value was used. In 4WG, Mauchly test was valid for all directions. If the Mauchly test was violated (*p* < 0.05), an additional Epsilon test was assessed. If the Epsilon was greater than 0.75, the Greenhouse–Geisser correction was used to assess for significance (*p* < 0.05). Abbreviations: SD, standard deviation; FU, Follow-Up; ABD, abduction; FLEX, forward flexion; ER, external rotation; IR, internal rotation.

4 WG		Mean	SD	Mauchly	*p*-Value
Direction	FU				
ABD				0.543	<0.001
	6 weeks	70	23		
	3 months	93	27		
	1 year	113	37		
FLEX				0.525	<0.001
	6 weeks	77	27		
	3 months	107	28		
	1 year	122	31		
ER				0.449	<0.001
	6 weeks	14	11		
	3 months	23	17		
	1 year	35	9		
IR				0.442	0.008
	6 weeks	71	22		
	3 months	76	15		
	1 year	85	16		

**Table 5:** Changes in scores at the follow-up examinations within for the 4-week group. Data are shown as mean ± standard deviation. A repeated measures ANOVA was performed for the ASES, SST, and VAS. If the Mauchly test was valid (*p* > 0.05), sphericity was assumed and the Mauchly *p*-value was used. The Mauchly test was valid for all ROM. For the SWB score as an ordinal scale, the Friedman test was employed, as marked in cursive. Abbreviations: ASES, American Shoulder and Elbow Surgeons Shoulder score; SST, Simple Shoulder Test score; VAS, Visual Analog Scale; SWB, subjective well-being.

4 WG		Mean	SD	Mauchly	*p*-Value
Score	FU				
ASES				0.088	<0.001
	6 weeks	53.7	9.8		
	3 months	64.6	13.3		
	1 year	83.5	16.1		
SST				0.054	<0.001
	6 weeks	2.6	1.3		
	3 months	4.0	2.3		
	1 year	8.0	3.2		
VAS				0.133	0.291
	6 weeks	0.8	1.3		
	3 months	0.8	1.2		
	1 year	0.4	1.0		
SWB				Friedman	0.107
	6 weeks	0.3	0.5		
	3 months	0.6	0.9		
	1 year	0.4	0.8		

**Table 6:** Changes in scores at the follow-up examinations within for the 6-week group. Data are shown as mean ± standard deviation. A repeated measures ANOVA was performed for the ASES, SST, and VAS. If the Mauchly test was valid (*p* > 0.05), sphericity was assumed and the Mauchly *p*-value was used. The Mauchly test was valid for all scores. For the SWB score as an ordinal scale, the Friedman test was employed, as marked in cursive.

6 WG		Mean	SD	Mauchly	*p*-Value
Score	FU				
ASES				0.530	<0.001
	6 weeks	56.6	11.2		
	3 months	56.9	12.1		
	1 year	80.1	12.1		
SST				0.956	<0.001
	6 weeks	2.1	1.2		
	3 months	3.8	1.9		
	1 year	7.1	2.1		
VAS				0.691	<0.001
	6 weeks	2.1	1.4		
	3 months	1.5	1.1		
	1 year	0.6	1.2		
SWB				Friedman	<0.001
	6 weeks	0.7	0.5		
	3 months	1.1	0.6		
	1 year	0.4	0.8		

Abbreviations: SD, standard deviation; ASES, American Shoulder and Elbow Surgeons Shoulder score; SST, Simple Shoulder Test score; VAS, Visual Analog Scale; SWB, subjective well-being.

## Data Availability

Dataset available on request from the authors.
